# Live Intracellular Biorthogonal Imaging by Surface Enhanced Raman Spectroscopy using Alkyne-Silver Nanoparticles Clusters

**DOI:** 10.1038/s41598-018-31165-3

**Published:** 2018-08-23

**Authors:** Matteo Ardini, Jian-An Huang, Carlos S. Sánchez, Mansoureh Z. Mousavi, Valeria Caprettini, Nicolò Maccaferri, Giovanni Melle, Giulia Bruno, Lea Pasquale, Denis Garoli, Francesco De Angelis

**Affiliations:** 1 Istituto Italiano di Tecnologia, Via Morego 30, 16163 Genova, Italy; 2 INCLIVA Instituto de Investigación Sanitaria, Av. Menéndez Pelayo 4, 46010 Valencia, Spain; 3 University of Genova, Via Balbi 5, 16126 Genova, Italy; 4 AB ANALITICA s.r.l., Via Svizzera 16, 35127 Padova, Italy

## Abstract

Live intracellular imaging is a valuable tool in modern diagnostics and pharmacology. Surface Enhanced Raman Spectroscopy (SERS) stands out as a non-destructive and multiplexed technique, but intracellular SERS imaging still suffers from interfering background from endogenous components. Here we show the assembly of small colloidal SERS probes with Raman signal in the cell-silent window of 1800–2900 cm^−1^ for biorthogonal intracellular SERS imaging of dopamine that was undistinguishable from the endogenous cell background. By linking colloidal silver nanoparticles with alkyne-dopamine adducts, clusters are formed by 2–6 nanoparticles spaced by tight interparticle gaps that exhibited high electric field enhancement and strong SERS signals of alkyne and dopamines. Due to the cell-silent signals of the alkyne, intracellular *in-vitro* Raman imaging shows that the dopamines on the internalized clusters remain distinguishable across the cytoplasm with good spatial resolution. Our method can be a general-purpose method for real-time imaging of biomolecules, such as proteins, peptides, DNA and drugs.

## Introduction

Cell’s life is characterized by many tangled molecular processes whose lack of regulation might lead to damages or diseases. Investigating these processes while keeping healthy cells could thus allow reliable detection of aberrations with no experimental artifacts. This perspective is under the limelight for modern diagnostic and pharmacological purposes though comes with a huge cost as all current cell-imaging techniques such as bright field, electron and atomic force microscopy as well as mass spectrometry, are unsuitable to carry out live analysis. For instance, they lack of satisfactory spatial resolution to scan the crowded cell environment or need for heavy treatments such as fixation, which lead to cell death. Even if considering fluorescence microscopy some drawbacks still remain, *e.g*. the limited number of labeled targets being detected simultaneously as well as the risk of photobleaching or impairment of the targets’ properties after labeling^1,2^.

In this context, the Raman spectroscopy is rising up as a cutting-edge technique to achieve accurate non-destructive live-cell imaging. Indeed, it is insensitive towards water-containing samples and has no need for molecular labels while exhibiting 1 µm resolution, which adequately fits with the size of the cell’s sub-compartments thus allowing collecting comprehensive information. Such a potential is related to the target molecules being detected, mainly molecular structure and bond type, which give rise to a spontaneous “fingerprint” scattering of the light when exposed to a laser. With no exceptions, all medically interesting molecules such as proteins, peptides, nucleic acids, neurotransmitters and drugs, exhibit their own scattering becoming distinguishable across the cell^1–3^. By virtue of this, several studies have been carried out unveiling the molecular details of the mechanisms ruling the cell’s life, *e.g*. ionic transport across the membrane^4^ and the transitions between cell cycle phases^5^ as well as cancers classification^6^ and identification^7^.

**Figure 1:**
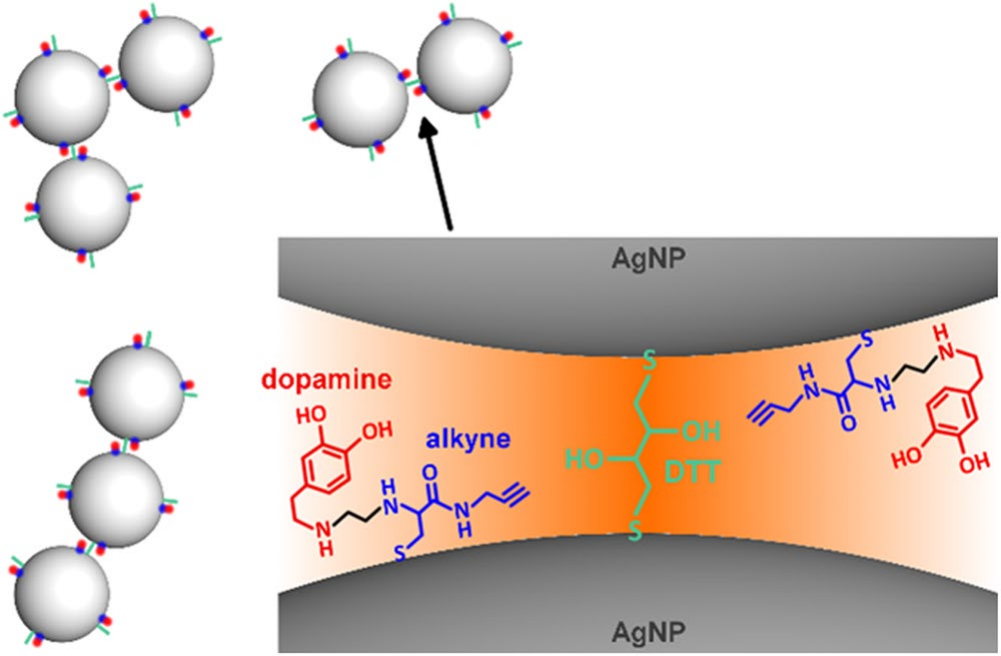
Cluster’s base structure. The silver-sulfur interaction is exploited to link alkyne-dopamine adducts and DTT molecules, both bearing a free –SH group, on the surface of AgNPs. DTT also provides a second –SH to trigger assembly of nanoparticles into small colloidal clusters. The DTT layer ensures tailoring of nano-sized interparticle gaps where SERS rises up to enhance the Raman detectability of the alkyne-dopamine adducts in the cell-silent window upon inside the cell.

Though overcoming the limits inherent in traditional cell imaging techniques, intracellular Raman spectroscopy introduces some issues. First, biomolecules suffer of weak scattering thus requiring high power lasers and/or long exposure times to be detected. Furthermore, cells typically exhibit a jumbled Raman spectrum due to the myriad of the endogenous biomolecules making targets detection and data interpretation very demanding^8^. To address these issues, new smart strategies have been recently developed^9^ such as the biorthogonal one where data acquisition is focused on the “cell-silent window” of the Raman spectrum, *i.e*. 1800–2900 cm^−1^, where the background scattering from native cell’s components is pretty much zeroed. To this aim, biorthogonal Raman tags have been obtained such as the alkyne derivatives whose C≡C triple bonds never belong to cells and exhibit scattering typically between 2000 and 2250 cm^−1^ ^9,10^. Since the first demonstration^10^, this strategy has been extensively reported for live intracellular imaging^11–20^. In this paper, SERS active plasmonic nanometric clusters are exploited for *in-vitro* intracellular detection through an alkyne-based biorthogonal strategy. To this aim, clusters are obtained via wet chemistry where colloidal 10–20 nm AgNPs (silver nanoparticles) are functionalized with alkyne-dopamine adducts before assembling into small colloidal assemblies using DTT (dithiothreitol) as crosslinking agent (Fig. 1). Nanoparticles functionalization and crosslinking are achieved through –SH (thiol) groups of the alkyne-dopamine and DTT moieties, whose high affinity towards noble metals is well known^21–23^. By means of visible and Raman spectroscopy, electron microscopy, dynamic light scattering and numerical simulations, it is shown that 25 to 120 nm AgNPs + alkyne-dopamine + DTT clusters are obtained and readily internalized within fibroblast cells likely due to an endocytosis mechanism and driven to gather into clearly distinguishable hot spots within the cytosol. The clusters exhibit a remarkable plasmonic behavior, mostly rising from the interparticle gaps between the single building nanoparticles, which greatly enhances the detectability of the alkyne within the cell-silent window, otherwise too weak for detection^14,20^. Such an effect is useful if considering that the other exogenous biocompatible tag, *i.e*. dopamine, still possesses scattering overlapping that Raman active region of cells thus remaining undistinguishable even with the aid of the plasmonic enhancement. In addition, the clusters also exhibit long colloidal stability outside cells and apparent biocompatibility after endocytosis, which make them suitable for attachment of other different (bio)molecules.

## Results and Discussion

### Optical properties

**Figure 2:**
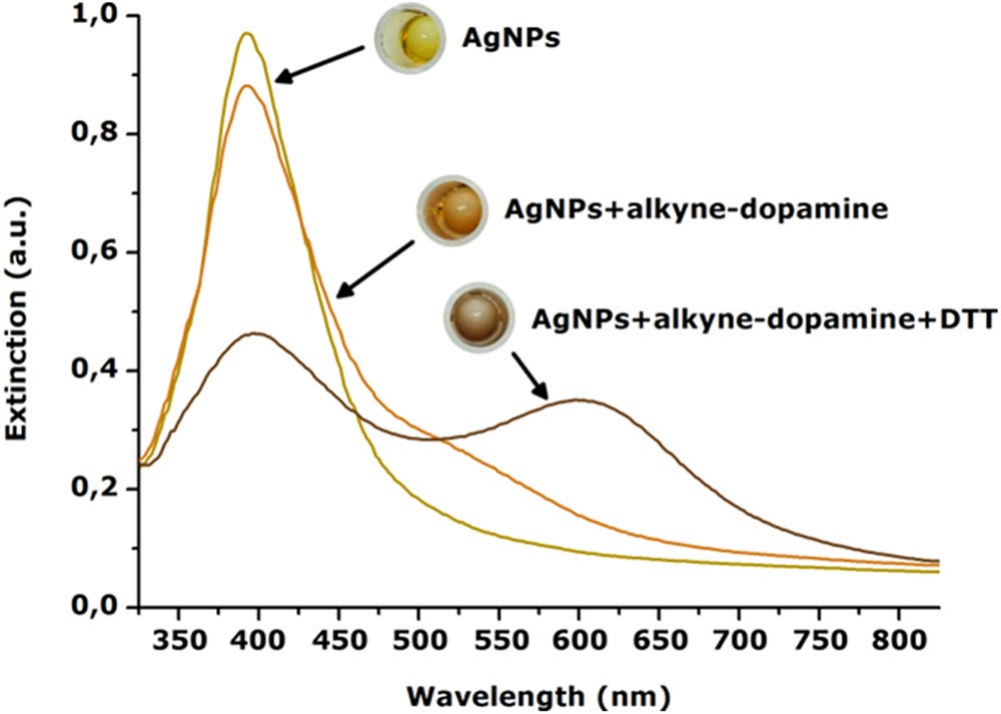
Extinction spectra of AgNPs before and after reaction with alkyne-dopamine and DTT. The 400 nm resonance peak of AgNPs shows a shoulder around 520 nm upon interaction with the –SH-containing alkyne-dopamine adducts likely due to metal surface functionalization. Upon reacting with DTT, carrying a couple of –SH, a second distinguishable broad peak is detected around 600 nm suggesting clustering of the nanoparticles. These optical transitions are even distinguishable by eye inspection as the color turns from yellow to brown.

Before reaction with the acid bis(N-hydroxysuccinimide ester)-crosslinked alkyne-dopamine adducts, the colloidal solution of AgNPs showed a yellow color and a fairly sharp extinction spectrum with a resonance peak positioned at 400 nm which typically belongs to small (10–20 nm) and unfunctionalized (citrate-coated) nanoparticles (Fig. 2, yellow spectrum). Following the incubation with the alkyne-dopamine adducts a fast color change occurred from yellow into a light orange shade likely meaning that the metal surface underwent functionalization. Consequently, the resulting extinction spectrum produced a little though well distinguishable shoulder to higher wavelengths centered at 520 nm while keeping the 400 nm peak as the main signal (Fig. 2, orange spectrum). Such an optical transition was likely due to the establishment of new stable bonds occurring between the silver surface and the free –SH group unequivocally belonging to the alkyne tag. As a matter of fact, no changes were obtained if incubating AgNPs with a alkyne-free solution of suberic acid bis(N-hydroxysuccinimide ester) and dopamine. Further optical transition occurred upon mixing the AgNPs + alkyne-dopamine species with DTT. This produced a quick color change to brown resulting in the appearance of a well distinguishable second broad extinction peak around 600 nm (Fig. 2, brown spectrum). This suggested that a new heterogeneous population of species formed reasonably due to the clustering of AgNPs + alkyne-dopamine adducts into larger assemblies, as also reported in other studies^24–27^. This was likely due to the crosslinking role of DTT carrying a double –SH group capable to simultaneously bind two nanoparticles. Similar results have been obtained for DTT-crosslinked assemblies of gold nanoparticles^28^. Interestingly, the AgNPs + alkyne-dopamine + DTT sample remained stable by several days at 4 °C without undergoing excessive precipitation. We have been able to exploit such an effect by adding DTT to pre-assembled AgNPs + alkyne-dopamine adducts to final molar ratio 264:1 (DTT:adducts) to trigger clustering. This amount was found to properly guarantee instantaneous clustering while keeping the formed assemblies water-soluble and stable without undergoing precipitation. Both these effects have been ascribed to the DTT moieties. Namely, expect those moieties whose thiols couples were fully engaged in crosslinking the nanoparticles, the remaining ones possessed a free –SH which is dissociated to negatively charged thiolate, –S^−^, due to its acid behavior (pK_a_ = 9.2 or 10.1) compared to the aqueous solution (pK_a_ = 14). This layer of thiolates thus was likely to provide the silver surface with a net negative charge as a whole guaranteeing repulsion between the assembled clusters. On the other hand, the alkyne-dopamine layer assembled on AgNPs before the addition of DTT would provide only negligible effect on clustering because of their single –SH functionality.

**Figure 3:**
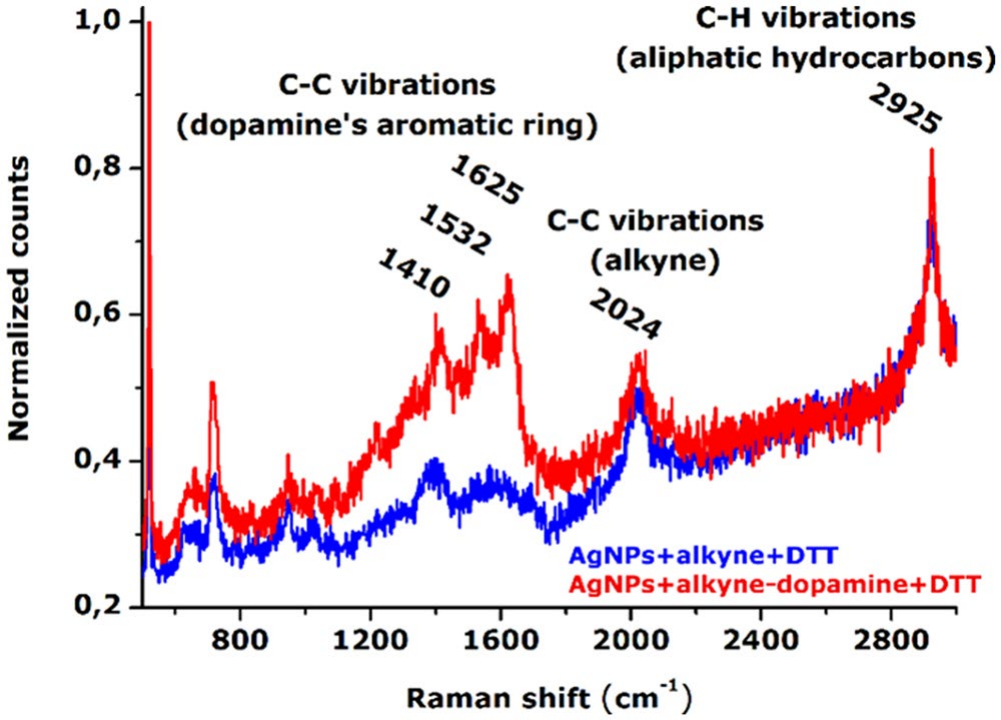
Raman spectra of AgNPs after reaction with alkyne-dopamine and DTT. The alkyne’s Raman scattering is detected at 2024 cm^−1^ when attached on AgNPs due to its C≡C triple bond. A second peak falls at 2925 cm^−1^ being ascribable to the C–H stretching of the aliphatic hydrocarbon chain. When including the dopamine moiety linked to the alkyne, the sample exhibits three additional peaks positioned at 1410, 1532 and 1625 cm^−1^ which belong to the aromatic C–C vibrations of the dopamine’s benzene ring.

The light scattering activity of the AgNPs + alkyne-dopamine + DTT sample was investigated by Raman spectroscopy. Firstly, control samples of aqueous solutions containing AgNPs, alkyne, dopamine, DTT, suberic acid bis(N-hydroxysuccinimide ester) or PVA were analyzed without giving appreciable signals in a huge wavelength range from 500 to 3000 cm^−1^. Conversely, the AgNPs + alkyne + DTT species showed two distinct sharp peaks positioned at 2024 cm^−1^ and 2925 cm^−1^. The 2024 cm^−1^ peak is known to belong to the C≡C triple bond^14^ thus undoubtedly ascribable to the alkyne moiety covalently linked to the silver surface. The 2925 cm^−1^ belongs to the C–H stretching vibrations of aliphatic hydrocarbon chains^29^ thus ascribable to the alkyne’s hydrocarbon chain in this case (Fig. 3, blue spectrum). Similarly, the AgNPs + alkyne-dopamine + DTT sample exhibited both the peaks at 2024 and 2925 cm^−1^ which were ascribed to the alkyne tag. In addition, three more sharp signals positioned at 1410, 1532 and 1625 cm^−1^ were also detected (Fig. 3, red spectrum). The Raman peaks observed in the wavelength range 1400–1650 cm^−1^ are known to belong to the C–C vibrations of the aromatic benzene ring^29^. In this case, the aromatic benzene ring was unequivocally found on the dopamine molecule (see Fig. 1 for alkyne’s and dopamine’s molecular structures). To note, the presence of dopamine covalently linked to the alkyne did not induced wavelength shift of the alkyne’s scattering as one could expect from previous studies^14^, probably because of the long distance between dopamine and the C≡C triple bond. These data proved the presence of both alkyne and dopamine on the silver surface as expected because of the crosslinking reaction used to bind the molecules. These were pivotal results as the spontaneous scattering of free dopamine and alkyne is very negligible in the absence of AgNPs as long as their concentrations are below hundreds mM, that would be obviously unsuitable to ensure healthy physiological conditions for cells.

### Morphological and topographical features

**Figure 4:**
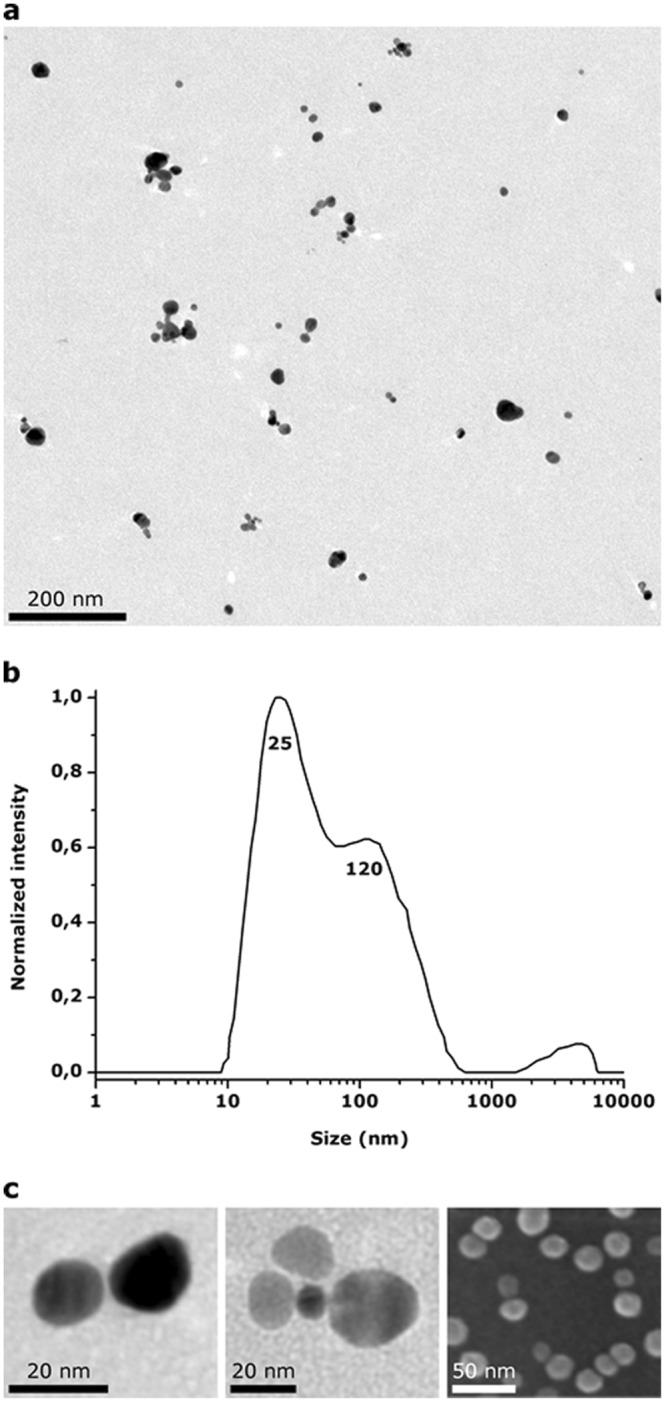
Structural features of AgNPs after reaction with alkyne-dopamine and DTT. (**a**) TEM low-magnification micrograph showing a heterogeneous population of single and clustered nanoparticles. Clusters are mostly made by 2–6 AgNPs. (**b**) Dynamic light scattering analysis showing two main populations with 25 and 120 nm hydrodynamic diameters. (**c**) TEM and SEM high-magnification images showing the interparticle gaps within the clusters. Gaps appear 1–2 nm wide. The thin organic monolayers of alkyne-dopamine and DTT cannot be clearly observed.

The structural features of the AgNPs + alkyne-dopamine + DTT sample have been investigated by TEM and SEM. Specimens were obtained by dropping and drying a fresh sample solution on carbon-coated grids or gold-coated microscope coverslips before washing with ultra-pure water and additional drying. The resulting micrographs are shown in Fig. 4. As a first result, several clusters species were found out all over the grid with also some single nanoparticles lying in-between. As expected, according to the extinction spectrum showing a broad double peak trend (Fig. 2, brown line), clusters appeared heterogeneously dispersed and typically made by 2 to 6 AgNPs tightly linked each other meaning that the crosslinking role of DTT occurred during the assembly process (Fig. 4a). In addition, some rare large-sized clusters (>1000 nm) made by multiple nanoparticles were also found reasonably as a side product of the process. Supporting data were obtained by dynamic light scattering analysis which confirmed the size range of the clusters found by TEM showing a heterogeneous population with two main peaks corresponding to hydrodynamic diameters of 25 to 120 nm, respectively (Fig. 4b). Notably, as the clusters were horizontally lying on the carbon substrate, the interparticle gaps between two adjacent single AgNPs became clearly visibly at high-magnification micrographs. According to the thickness of the alkyne-dopamine and/or DTT self-assembled monolayer over the metal surface, these gaps were found to be 1–2 nm wide thus theoretically suitable in creating a plasmonic coupled electric filed (Fig. 4c).

**Figure 5:**
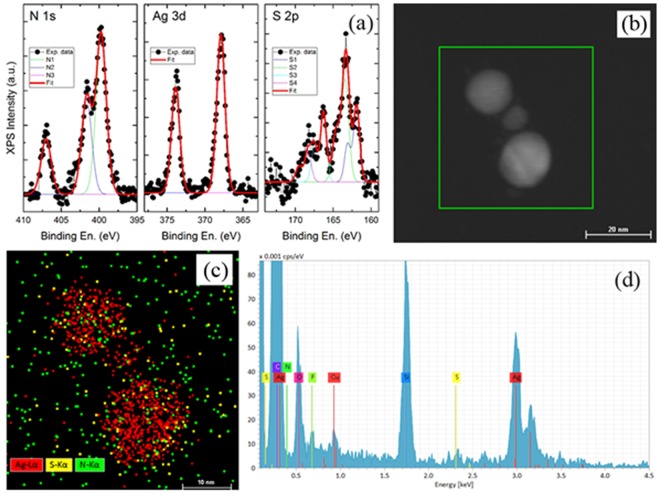
Elemental analysis of the AgNPs after reaction with alkyne-dopamine and DTT. (**a**) N 1s, Ag 3d and S 2p XPS data, together with the results of the best fit procedure; (**b**) TEM micrograph of 3 NPs cluster; (**c**) STEM-EDS map; (**d**) EDS spectrum.

Additional the elemental analyses of clusters have been carried out by EDS and XPS. In all the cases, spectrum showed the presence of the main elements making the clusters, hence confirming the actual functionalization of the system. Figure 5a illustrates the results of the XPS characterization (additional details on the analysis are reported in Supporting Informations) over the energy regions typical for N 1s, Ag 3d and S 2p peaks. Silver signals are consistent with the presence of metallic Ag nanoparticles, Nitrogen spectrum is clearly composed of at least three different components, centered at ~399.7 eV (N1 component), ~401.7 eV (N2 component) and ~407.0 eV (N3 component). N1 component therefore could be assigned to secondary amine groups in both the “alkyne” molecule chain and bound “dopamine-alkyne”, while N2 component could represent a fraction of unbound dopamine molecules. N3 component, instead, is found at a position that is typical of nitrate (−NO3) groups. Finally the Sulphur; the best fit was obtained considering four different S components. The positions of the four S components (considering for each of them the position of the most intense component of the doublet) are ~161.9 eV (S1 component), ~163.4 eV (S2 component), ~166.3 eV (S3 component) and ~168.3 eV (S4 component). The position of the S1 component is consistent with the formation of a thiol-Ag bond^30^, and therefore its presence confirms the effective functionalization of our Ag particles with the used ligand molecules. The position of the S2 component is consistent with results reported on free or unbound thiols, while components S3 and S4 are centered at positions that are typical of oxidized sulphur species, as sulfones. Figure 5b reports an example of STEM-EDS analysis (additional ones are reported in Supporting Information) also confirming the presence of silver actually functionalized with the organic compounds previously described.

**Figure 6:**
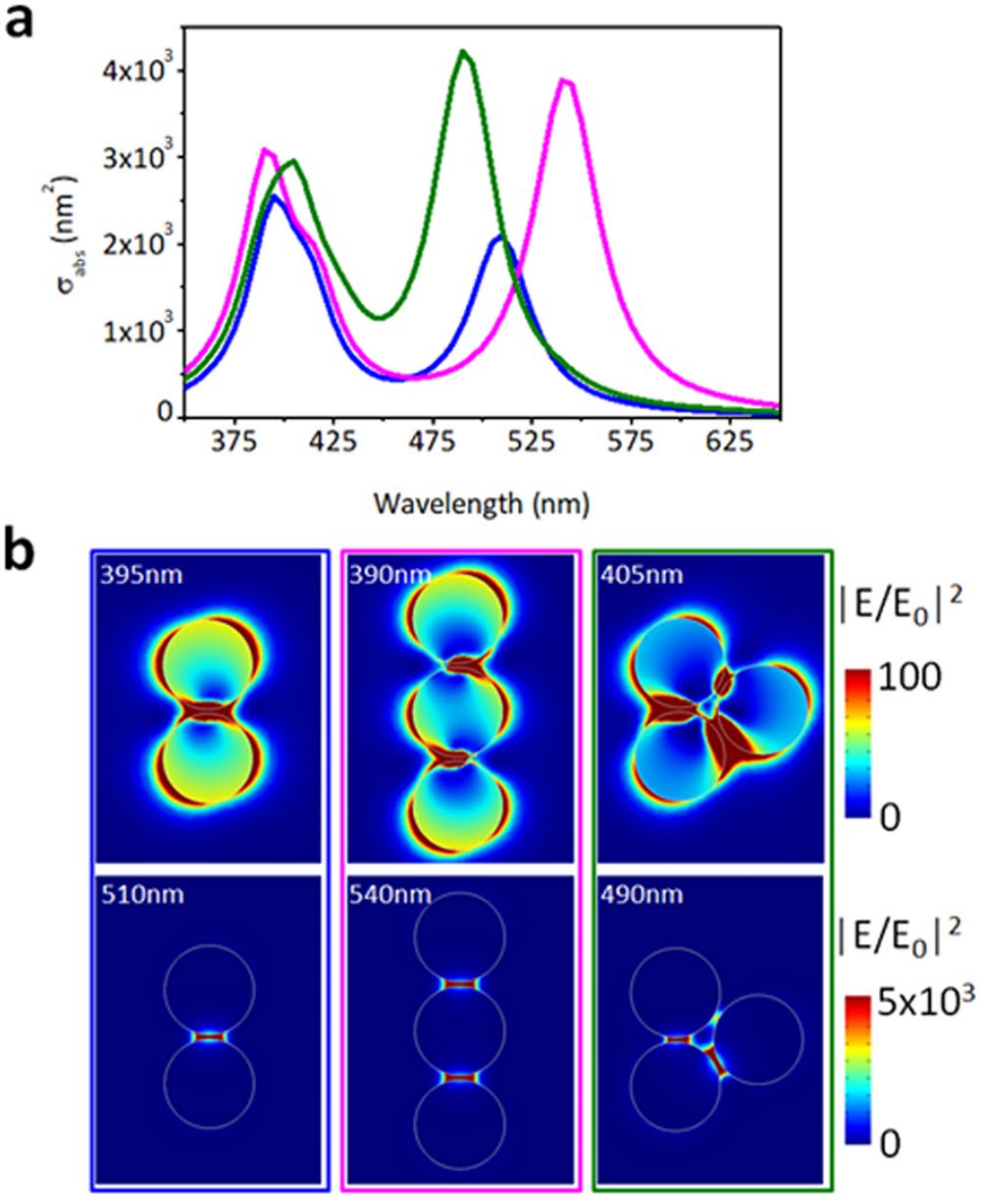
Numerical simulation of the far- and near-field optical response of a AgNP-AgNP dimer and trimer. (**a**) Extinction efficiency of the three systems considered for the case of an incident unpolarized electric field. Two resonances can be seen, one related to the single particle at around 400 nm and the other one related to the near-field coupling between the two particles at 510 nm and between the three particles at 540 nm or 490 nm depending on the relative orientation between the particles. (**b**) Near-field intensity distribution for the single particle mode (top-panels) and the dimer resonance (bottom-panels).

To get more physical insights of the far- and near-field optical response of the smallest possible cluster, that is an isolated AgNP-AgNP dimer, numerical simulations have been carried out. As the real sample consists of a solution of many and randomly oriented dimers, the correct optical response can be retrieved by summing up the effects of the fundamental configurations shown in Fig. 6. According to the extinction spectra obtained from the AgNPs before inducing clustering (Fig. 2), the first peaks at around 400 nm are mainly due to the excitation of the single AgNP spheres dipolar localized surface plasmon resonances (Fig. 6 top-panels), although they interact a bit between them due to the unpolarized nature of the incident light. The second peaks between 490 nm and 540 nm nm are due to the excitation of the hybrid bonding modes of the clusters, which is most likely a dimer or a trimer (Fig. 6b bottom-panels), where a strong near field enhancement of more than 5000 is reached. This implies a field intensity enhancement of almost 6 × 10^4^ in a very small volume (<10 nm^3^). The thiol-mediated coating of DTT over the metal surface was supposed to be the key driving force inducing the plasmonic behavior of the assembled clusters. High-magnification electron micrographs proved that clusters formed upon assembly were endowed with very tight interparticle gaps between the single AgNPs building blocks where strong confinement of electric field was supposed to rise (Fig. 3c). The dimer and trimer models were chosen as reference configuration to demonstrate the putative plasmonic behavior of the clusters. These configurations are actually the best ones to enhance the optical response in a small volume and it has been used in the past also to increase the sensitivity of detection of molecular binding events^31^, as well as to obtain structural and functional information on nanoscale distances^32^.

### Intracellular Raman spectroscopy

**Figure 7:**
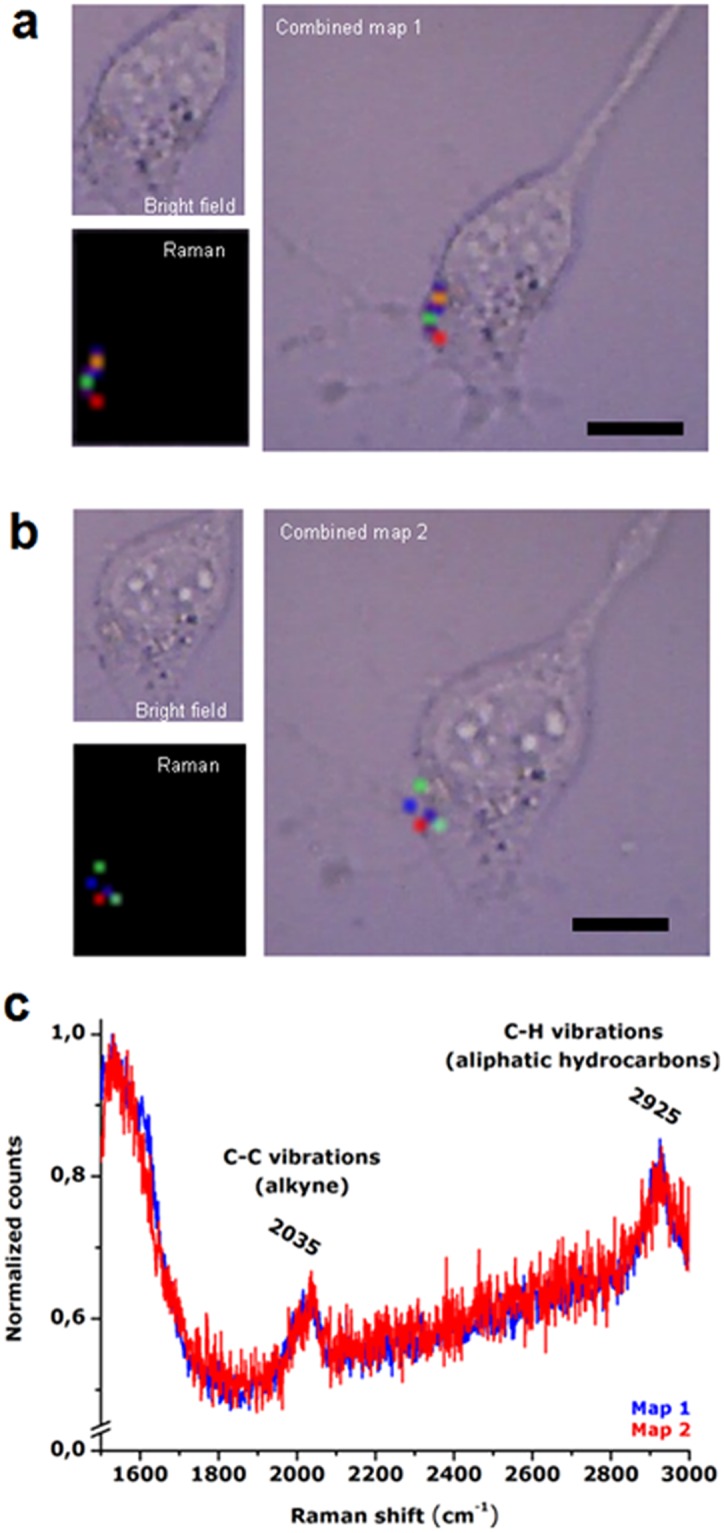
Intracellular Raman imaging on fibroblasts after endocytosis of clusters. (**a**) Raman map of AgNP dimers is observed within the fibroblasts’ cytoplasm as distinguishable randomly dispersed hot spots (highlighted by color dots related to Raman intensity of the alkyne 2035 cm^−1^ band). (**b**) After multiple imaging scans fibroblasts start showing volume swelling though still showing internalized hot spots. All scalebars are 10 µm. (**c**) Raman spectra extracted from the hot spots showing two main signals in the cell-silent window positioned at 2035 and 2925 cm^−1^ which are ascribable to the alkyne tag thus indicating the presence of the alkyne-linked dopamine inside cells.

Endocytosis of the AgNPs + alkyne-dopamine + DTT clusters was investigated by intracellular Raman analysis under *in-vitro* conditions based on the alkyne tag’s scattering as the main tracking signal. Experiments were carried out on fibroblasts cultures grown 24 h on platinum-coated dishes with a diluted solution of freshly made clusters. Bright field micrographs of cells are shown in Fig. 7.

Visual inspection by bright-field microscopy showed good cell adhesion over the substrate meaning that cells did not suffer the growth conditions and the presence of metal clusters inside the medium. This result was quite encouraging as it is known the silver nanoparticles typically produce highly toxic conditions mostly derived from oxidative stress and DNA damage, which in turn lead to cell death. Therefore, it can be figured out that under these conditions clustering of the nanoparticles into negatively charged large complexes increased biocompatibility. Accordingly, it is reported that large-sized AgNPs exhibit less toxic-effect or even biocompatibility in various types of eukaryotes^33–36^.

In addition, no large clusters were visible indicating that during the incubation step with cells the metal did not undergo further clustering or excessive aggregation. Raman mapping of cells obtained by 1s scanning step using a 532 nm laser source produced multiple distinguishable hot spots within the cell volume. After background subtraction spots appeared located outside nuclei and randomly localized in the cytosol (Fig. 7a). In fact, this SERS map of alkyne suggested a map of AgNP dimers that exhibit strong plasmonic resonance at 532 nm as demonstrated by Fig. 6. To note, after multiple imaging scans by the laser source cells slowly started to show volume swelling that is known as a symptom of cell suffering caused by non-physiological conditions. However, under these conditions some hot spots were still detected (Fig. 7b). In both cases, the Raman spectra extracted from each single hot spot showed two distinct sharp peaks positioned at 2035 and 2925 cm^−1^ (Fig. 7c). The Raman background from native cell’s component was completely negligible in the region 1800–2900 cm^−1^ thus indicating that peaks were acquired in the silent window of the fibroblast, as expected. For this reason, detection of the dopamine’s scattering in the 1400–1650 cm^−1^ range was not taken into account due to the overlapping with the cell’s background. Due to the similarity of the spectrum recorded on the clusters solution before undergoing endocytosis (Fig. 3, red spectrum), both signals were ascribed to the alkyne tag meaning that the clusters were successfully internalized by fibroblasts. However, the low wavelength peak coming from the C≡C triple bond (2035 cm^−1^) observed after endocytosis resulted shifted by 11 cm^−1^ compared to that one observed before endocytosis, that is 2024 cm^−1^. On the other hand, the high wavelength peak at 2925 cm^−1^ was not affected. The wavelength shift of the alkyne’s scattering from 2024 to 2035 cm^−1^ observed before and after endocytosis, respectively, can be attributed to an inductive effect rising from the interaction of the C≡C triple bond with endogenous intracellular molecules floating in the cell volume, as supposed by published results^13,15^. Detection of alkyne within the cytoplasm indirectly proved the presence of dopamine being it covalently attached to the alkyne itself thus meaning that endocytosis of clusters occurred.

To date, there is still incomplete knowledge at the molecular level about the interactions between nanomaterials and biological systems. However, this phenomenon is under the limelight for being exploited for advanced nanomedicine purposes^37^. Typically, uptake of nanomaterials by cells is known to depends on their properties (shape, size, surface charge and chemistry) as well as those of cells (membrane fluidity, receptor density and number)^38^. As a rule, nanomaterials uptake occurs through passive or active. For our purpose, passive uptake was not likely to be the main path as nanoparticles can only enter if very small. Thus, active transport, that is endocytosis, was supposed to act better. Nowadays, endocytosis-mediated internalization of nanoparticles has been extensively explored unveiling that medium-sized nanoparticle (10 to 80 nm) with organic coatings and negative surface potential act as efficient players^39–44^. We accomplished this goal by exploiting 10–20 nm colloidal AgNPs as building blocks to gather into nanometric clusters taking advantage of DTT crosslinker (Fig. 4a). According to several studies^45–48^ and based on their size and negative charged surface, these probes were supposed to fit adequately with an endocytosis pattern to enter cells. The appearance of the 2034 cm^−1^ signal unequivocally indicated the presence of the alkyne tag and therefore of dopamine inside cells as the biomolecule was unlikely prone to be stripped from the cluster species due its covalent binding to the alkyne tag through the suberic acid bis(N-hydroxysuccinimide ester) crosslinker. Thus, embedding the exogenous cell-independent alkyne within a plasmonic nanostructure enabled us for easy imaging and gain of sensitivity to detect the dopamine target otherwise undetectable in a crowded environment such as the cytoplasm. Supporting these results, other examples of metal nanoarchitectures endowed with tight gaps between coupled plasmonic metal surfaces have been proposed and exploited as SERS-active probes for cell imaging^49–52^. Taking into account of the healthy conditions of cells observed after incubation with the clusters, cell swelling observed during Raman experiments could be associated some laser-effects such as heating. Alternatively, even oxidative stress triggered by dopamine oxidation may be considered as onset of impairment of mitochondria respiratory function, as showed in neuronal cells^53,54^. However, with respect to our purpose, we did not get insights of such effect though it can be figured out that improvement of the imaging conditions might be performed by using gold-based nanoclusters with different aspect ratios, for instance gold nanorods, with red-shifted surface plasmon resonances thus allowing the use of low-energy light sources.

## Conclusions

In this work, we have been successful in making exogenous nanometric probes, *i.e*. silver nanoclusters, detectable upon internalization in living cells by live-cell Raman spectroscopy by taking advantage of a biorthogonal tag as the alkyne’s C≡C bond chemically adsorbed on the metal surface. Since the peak appears in the Raman-silent window, distinguished detection of alkyne can be achieved regardless of background Raman signals from other molecular vibrations in the complex matrix. Visible and Raman spectroscopy, electron microscopy, dynamic light scattering and numerical simulations confirm the creation of plasmonic AgNPs + alkyne-dopamine + DTT clusters. The clusters with alkyne-dopamine adducts could work as a multiplexing SERS probe to trace the transport abnormalities of dopamines in neurons, the state-of-the-art tracking of which was done by fluorescent probes^55^. Biomolecules related to nervous system such as dopamine represent main targets in modern nanotechnology in that concerning real-time *in vivo* study of cell dynamics such as the synthesis and transport across cells. Consider for example the impact of dopamine transport and amount inside cells in promoting neurodegenerative diseases^56^. Thus, dopamine has been selected as biomedically interesting target to demonstrate that its detection inside living can be easily achieved using an alkyne probe. Otherwise the target cannot be detected within the cytoplasm.

Fibroblast cells internalize such clusters through endocytosis and drive them to gather into clearly distinguishable hot spots within the cytosol. Clusters are shown to possess plasmonic behavior and the ability to permeate cells without apparently causing suffering on cells. These results fit in a growing framework where modern drug discovery and targeted drug therapy is remarkably boosting the “live-cell imaging” market.

The synthesized clusters exhibit long colloidal stability outside cells and apparent biocompatibility after endocytosis. Such a simple strategy is versatile and virtually relevant for a wide range of other molecules. The clusters of AgNPs-alkyne tag can be incorporated with biologically important small biomolecules such as nucleic acids, proteins and drugs and can be used as a powerful tool for intracellular delivery of probes and targeting organelles, while simultaneously imaging the intercellular processes. This application could further be extended through commercially available alkyne derivatives with chemical functionalities such as amine-, carboxyl-, avidin- or thiol-reactive groups for fast biofunctionalization of proteins, antibodies and enzymes (see for instance amino-PEG4-alkyne, biotin-PEG4-alkyne alkyne-PEG5-N-hydroxysuccinimidyl ester reagents by Sigma-Aldrich). The possibilities can be further extended to multi-color bioimaging if considering that each alkyne tag has its own scattering wavelength while being detectable within the cell-silent window, as reported previously^12,14,20^.

## Methods

### Materials

A commercial solution of 0.1 mg·mL^−1^ colloidal citrate-coated AgNPs with diameter size 10–20 nm (3.78 nmol NPs per liter, 3.78 nM) were purchased from PlasmaChem GmbH. Powders of DTT, PVA (polyvinyl alcohol), dopamine hydrochloride and suberic acid bis(N-hydroxysuccinimide ester) and a solution of 99.9% DMSO (dimethyl sulfoxide) were purchased from Sigma-Aldrich. The alkyne derivative 2-amino-3-mercapto-N-(prop-2-ynyl)propionamide was used as Raman tag and purchased from AnaSpec, Inc. Low adsorption 1.5 mL tubes were purchased from Sigma-Aldrich. Cell cultures of NIH-3T3 fibroblasts were made in DMEM (Dulbecco’s Modified Eagle Medium) supplemented with 1% Pen/Strep (penicillin/streptomycin) antibiotics and 10% FBS (Fetal Bovine Serum) purchased from Sigma-Aldrich. Glass bottom culture dishes used for cells cultures were obtained from MatTek Corporation. Regenerated cellulose membranes for dialysis with 14 kDa cut-off were obtained from ThermoFisher Scientific. Carbon film-coated 200 mesh copper grids for electronic microscopy analysis were purchased from Nanovision S.r.l.

### Clusters assembly procedure

Clusters made by AgNPs, the alkyne Raman tag, dopamine and DTT were obtained by a wet chemistry procedure as follows. Firstly, three stock solutions of alkyne, dopamine hydrochloride and suberic acid bis(N-hydroxysuccinimide ester) were freshly made in 99.9% DMSO before sequentially mixing them up to final concentration of 3, 3 and 6 mM, respectively. The mixture was left reacting 1 hour at room temperature (20 °C) under 120 rpm orbital shacking. In this way, the covalent bond between alkyne and dopamine through their primary amines was accomplished through the amine-reactive homobifunctional crosslinker suberic acid bis(N-hydroxysuccinimide ester) to obtain hybrid alkyne-dopamine adducts. Meanwhile, the citrate-coated AgNPs were subjected to 10 s sonication in an ultrasonic water bath (Elma) at 20 °C by applying 59 kHz pulses with 100% power to break apart any preformed clusters. The so-treated AgNPs were mixed with 0.5% PVA chosen as an effective surfactant for silver, as reported previously^57^, before adding the alkyne-dopamine-suberic acid bis(N-hydroxysuccinimide ester) reaction to final AgNPs:adducts volume ratio of 150:1. Under these conditions, displacement of the citrate molecules by the alkyne-dopamine adducts occurred over the silver surface by ligand exchange. The so-obtained mix was incubated 30 min at room temperature under orbital shaking prior to add a fresh aqueous solution of DTT to final concentration of 1.3 µM followed by additional 30 min incubation. Finally, the mixture was thoroughly washed in milliQ water containing 0.5% PVA by exhaustive dialysis at room temperature to remove all small unbound reagents.

### Cell cultures

In order to acquire an enhanced Raman signal during intracellular imaging (see below), a 50 nm layer of 99.95% pure platinum was deposited on glass bottom culture dishes by sputter coating technique using a Q150T ES sputter (Quorum Technologies, Ltd). Afterwards, the platinum-coated dishes were sterilized by 20 minutes UV exposure in a laminar flow hood. Subsequently, fibroblast NIH-3T3 cells were seeded on the dishes at the density of 50·10^3^ cells per cm^2^ and grown in DMEM medium supplemented with 1% Pen/Strep and 10% FBS. Cells were incubated at 37 °C and 5% CO_2_ concentration for 24 h before replacing the medium with a fresh one supplemented with 1% Pen/Strep and 2% FBS previously mixed with a freshly synthesized solution of clusters at final medium:clusters volume ratio of 2:1. Under these conditions, the reduced concentration of serum does not let the formation of a protein corona that could inhibit the cellular uptake of nanoparticles by cells^58^. After 24 h incubation at 37 °C and 5% CO_2_ concentration, cells have been washed three times with phosphate buffer 1X to remove extracellular probes and directly analyzed by means of Raman spectroscopy (see below).

### Transmission and Scanning Electron Microscopy, Energy-Dispersive X-ray Spectroscopy and X-ray Photoelectron Spectroscopy

Morphological and topographical features of the assembled clusters were investigated by means of electron microscopy. To this aim, 3 μL of freshly prepared clusters solution were dropped on 200 mesh carbon film-coated grids and let completely dry under hood. Grids were then washed twice with milliQ water. Transmission Electron Microscopy (TEM) was performed by using a JEM 1011 microscope (JOEL) equipped with a tungsten filament operating at 80 keV. Complementary analysis were also performed by Scanning Electron Microscopy (SEM). In this case, specimens were made by dropping 2 μL of solution on microscope coverslips previously sputtered with 20 nm of gold, air dried under hood, washed with milliQ water and dried again with nitrogen. Analysis were performed using the electronic beam of a Helios Nanolab 650 Dual Beam (FEI) by applying a current of 0.2 nA and an acceleration of 5 kV. Images were taken in immersion mode using a TLD detector and collecting secondary electrons. If necessary, the obtained micrographs were processed using ImageJ v1.5i and/or GIMP v2.8.16 through FFT bandpass filter correction. To perform STEM-EDS analyses of Ag NP cluster, 3 μL of freshly prepared clusters solution were dropped on 150 mesh carbon film-coated grids, dried and washed with milliQ water. The analysis was performed with a FEI Tecnai G2 F20, operated at 200 kV, with a Bruker X-Flash 6|T 30 EDS silicon-drift detector (SDD). XPS analyses were carried out with a Kratos Axis UltraDLD spectrometer using a monochromatic Al Kα source (20 mA, 15 kV). The sample used for XPS characterization was drop casted on Au-coated silicon wafer. Survey scan analyses were carried out with an analysis area of 300 × 700 microns and a pass energy of 160 eV. High resolution analyses were carried out with an analysis area of 300 × 700 microns and a pass energy of 20 eV. The Kratos charge neutralizer system was used on all specimens. Spectra have been charge corrected to the main line of the carbon 1s spectrum (adventitious carbon) set to 284.8 eV.

### Absorbance and Raman spectroscopy

Absorbance spectra were acquired at room temperature by means of a microplate reader NanoQuant Infinite M200 spectrophotometer (Tecan) in the 300–900 nm wavelength range with scan step 2 nm while exciting 100 µL of sample solutions dropped within transparent 96-well plates (Thermo Scientific). Bright field images and Raman mapping images were acquired with a confocal unit Renishaw inVia Raman Microscope (Renishaw plc, UK). Raman experiments on clusters solutions were performed by exciting 5 µL of sample dropped on a 1 cm^2^ silicon dioxide substrate using a 532 nm laser with 3.06 mW power and 1s exposure time while acquiring using a 20X objective. For intracellular Raman imaging, experiments were carried out on fibroblast cells previously incubated 24 h with the clusters on platinum-coated dishes using a 532 nm laser with 1.60 mW power and 1s exposure time while acquiring with a 60X water immersion objective.

### Numerical simulations

Numerical simulations were carried out to investigate the putative optical and plasmonic behavior of the assembled clusters. To this aim, the electromagnetic response of an isolated (non-interacting) cluster (dimer or trimer) was simulated using the Finite-Element Method (FEM) implemented in the RF Module of Comsol Multiphysics®. The radius of the single sphere composing the dimer was set according to the average nanoparticles’ sizes obtained during TEM investigations, while the interparticle gap was set to 1 nm based on the length of a full-stretched DTT molecule. To consider the presence of the alkyne molecules, a dielectric shell with n = 1.44 and 1 nm thick was considered around each particle composing the dimer. The model computes the scattering, absorption, and extinction cross-sections of the dimer. The unit cell was set to be 150 nm wide in both x-, y- and z-directions, with perfect matching layers (200 nm thick) at the borders. An unpolarized plane wave impinges on the structure.

## Electronic supplementary material

Supporting Information
