# Author Correction: Live Intracellular Biorthogonal Imaging by Surface Enhanced Raman Spectroscopy using Alkyne-Silver Nanoparticles Clusters

**DOI:** 10.1038/s41598-018-33285-2

**Published:** 2018-10-23

**Authors:** Matteo Ardini, Jian-An Huang, Carlos S. Sánchez, Mansoureh Z. Mousavi, Valeria Caprettini, Nicolò Maccaferri, Giovanni Melle, Giulia Bruno, Lea Pasquale, Denis Garoli, Francesco De Angelis

**Affiliations:** 1 Istituto Italiano di Tecnologia, Via Morego 30, 16163 Genova, Italy; 2 INCLIVA Instituto de Investigación Sanitaria, Av. Menéndez Pelayo 4, 46010 Valencia, Spain; 3 University of Genova, Via Balbi 5, 16126 Genova, Italy; 4 AB ANALITICA s.r.l., Via Svizzera 16, 35127 Padova, Italy

Correction to: *Scientific Reports* 10.1038/s41598-018-31165-3, published online 23 August 2018

In this Article, the legend of Figure 6 is incorrect:

“Numerical simulation of the far- and near-field optical response of a AgNP-AgNP dimer and trimer. (a) Extinction efficiency of the three systems considered for the case of an incident unpolarized electric field. Two resonances can be seen, one related to the single particle at around 400 nm and the other one related to the near-field coupling between the two particles at 510 nm and between the three particles at 540 nm or 490 nm depending on the relative orientation between the particles. (b) Near-field intensity distribution for the single particle mode (top-panels) and the dimer resonance (bottom-panels).”

should read:

“Numerical simulation of the far- and near-field optical response of a AgNP-AgNP dimer and trimer. (a) Absorption cross section of the three systems considered for the case of an incident unpolarized electric field. Two resonances can be seen, one related to the single particle at around 400 nm and the other one related to the near-field coupling between the two particles at 510 nm and between the three particles at 540 nm or 490 nm depending on the relative orientation between the particles. (b) Near-field intensity distribution for the single particle mode (top-panels) and the dimer resonance (bottom-panels).”

