# Supplementary material for: Live Intracellular Biorthogonal Imaging by Surface Enhanced Raman Spectroscopy using Alkyne-Silver Nanoparticles Clusters
Source: Sci Rep. 2018 Aug 23;8:12652. doi: 10.1038/s41598-018-31165-3 (PMC6107644; doi:10.1038/s41598-018-31165-3)
Supplement: Supplementary file 1 — Supporting Information [file 41598_2018_31165_MOESM1_ESM.docx]

**SUPPORTING INFORMATION**

**Live Intracellular Biorthogonal Imaging by Surface Enhanced Raman Spectroscopy using Alkyne-Silver Nanoparticles Clusters**

*Matteo Ardini^a^, Jian-An Huang^a^, Carlos S. Sánchez^b^, Mansoureh Z. Mousavi^a^,Valeria Caprettini^a,c^, Nicolò Maccaferri^a^, Giovanni Melle^a,c^, Giulia Bruno^a,c^, Lea Pasquale^a^, Denis Garoli^a,d*^, and Francesco De Angelis^a^**

^a^Istituto Italiano di Tecnologia, Via Morego 30, 16163 Genova, Italy

^b^INCLIVA Instituto de Investigación Sanitaria, Av. Menéndez Pelayo 4, 46010 Valencia, Spain

^c^University of Genova, Via Balbi 5, 16126 Genova, Italy

^d^AB ANALITICA s.r.l., Via Svizzera 16, 35127 Padova, Italy

**Corresponding author:** [francesco.deangelis@iit.it](mailto:francesco.deangelis@iit.it); denis.garoli@iit.it

**Supporting note 1:** XPS analysis

Fig. 5a in the main manuscript reports the results of the XPS characterization over the energy regions typical for N 1s, Ag3d and S 2p peaks, after subtraction of a Shirley type background. The figure reports as well the results of peak decomposition, that could be summarized as follows. Silver signals (a doublet of peaks, with the main component centered at ̴367.9 eV) are consistent with the presence of metallic Ag nanoparticles, in agreement with results reported in reference [Supporting Ref. 1]. Nitrogen spectrum is clearly composed of at least three different components, centered at ̴399.7 eV (N1 component), ̴401.7 eV (N2 component) and ̴407.0 eV (N3 component). N1 and N2 component positions are in close agreement with values reported in supporting ref. [2] for R−NH−R and R−NH2 moieties, respectively. N1 component therefore could be assigned to secondary amine groups in both the “alkyne” molecule chain and bound “dopamine-alkyne”, while N2 component could represent a fraction of unbound dopamine molecules. N3 component, instead, is found at a position that is typical of nitrate (-NO3) groups [Supporting Ref. 1]. Coming to sulphur, the best fit was obtained considering four different S components, each of those is represent by a spin-orbit coupled doublet, showing a branching ratio of 2:1 and a doublet splitting of 1.2 eV. The positions of the four S components (considering for each of them the position of the most intense component of the doublet) are ̴161.9 eV (S1 component), ̴163.4 eV (S2 component), ̴166.3 eV (S3 component) and ̴168.3 eV (S4 component). The position of the S1 component is consistent with the formation of a thiol-Ag bond [main text ref. 59], and therefore its presence confirms the effective functionalization of our Ag particles with the used ligand molecules. The position of the S2 component is consistent with results reported on free or unbound thiols, while components S3 and S4 are centered at positions that are typical of oxidized sulphur species, as sulfones [Supporting Ref. 1].

**Supporting note 2:** STEM – EDS analyses

Figure S1, S2 and S3 report other examples of STEM – EDS maps performed on AgNPs clusters. From the figures it can be easily demonstrated the presence of all the elements expected.


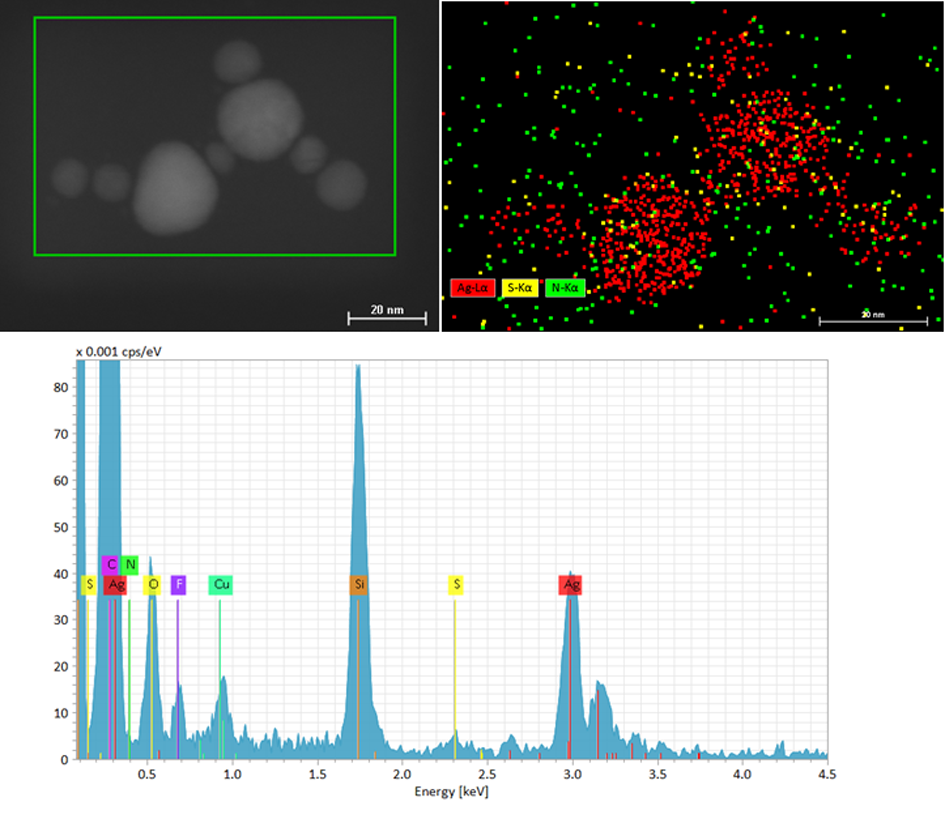


**Supporting Figure 1.** STEM-EDS Map of 8 functionalized AgNps in cluster.


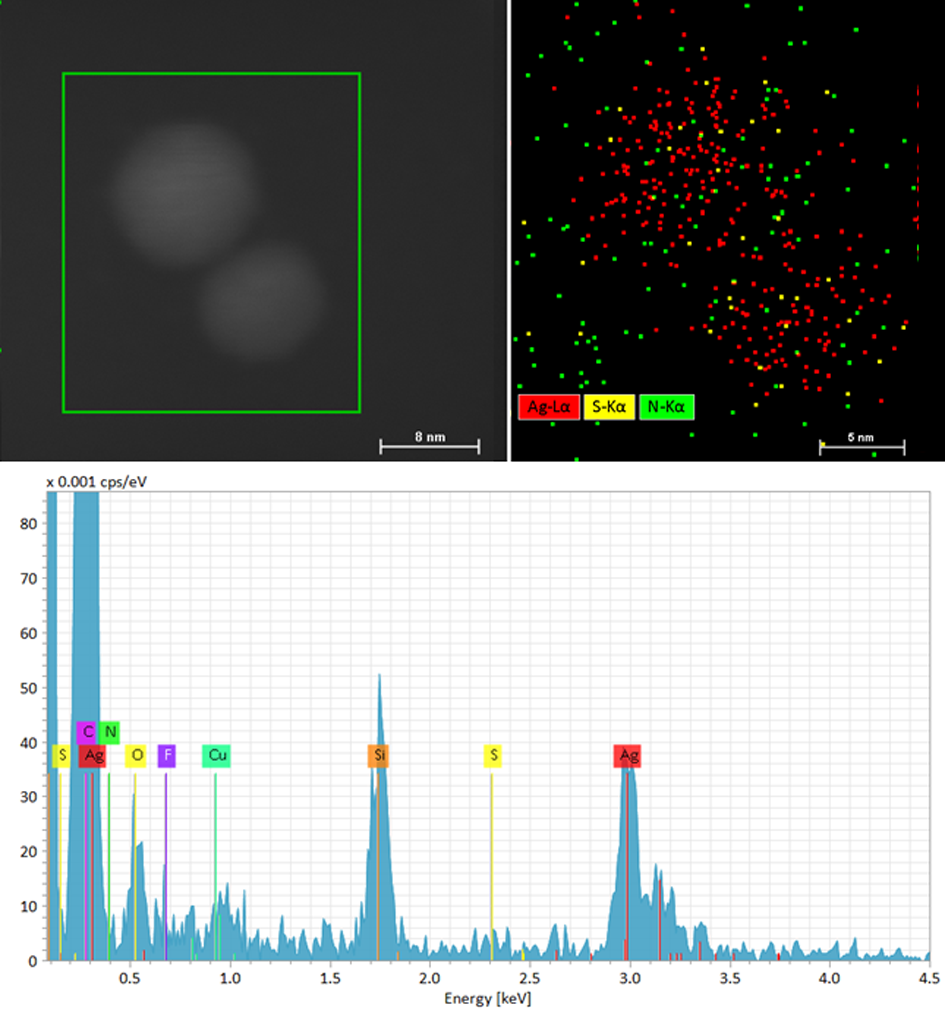


**Supporting Figure 2.** STEM-EDS Map of 2 functionalized AgNps in a dimer configuration.


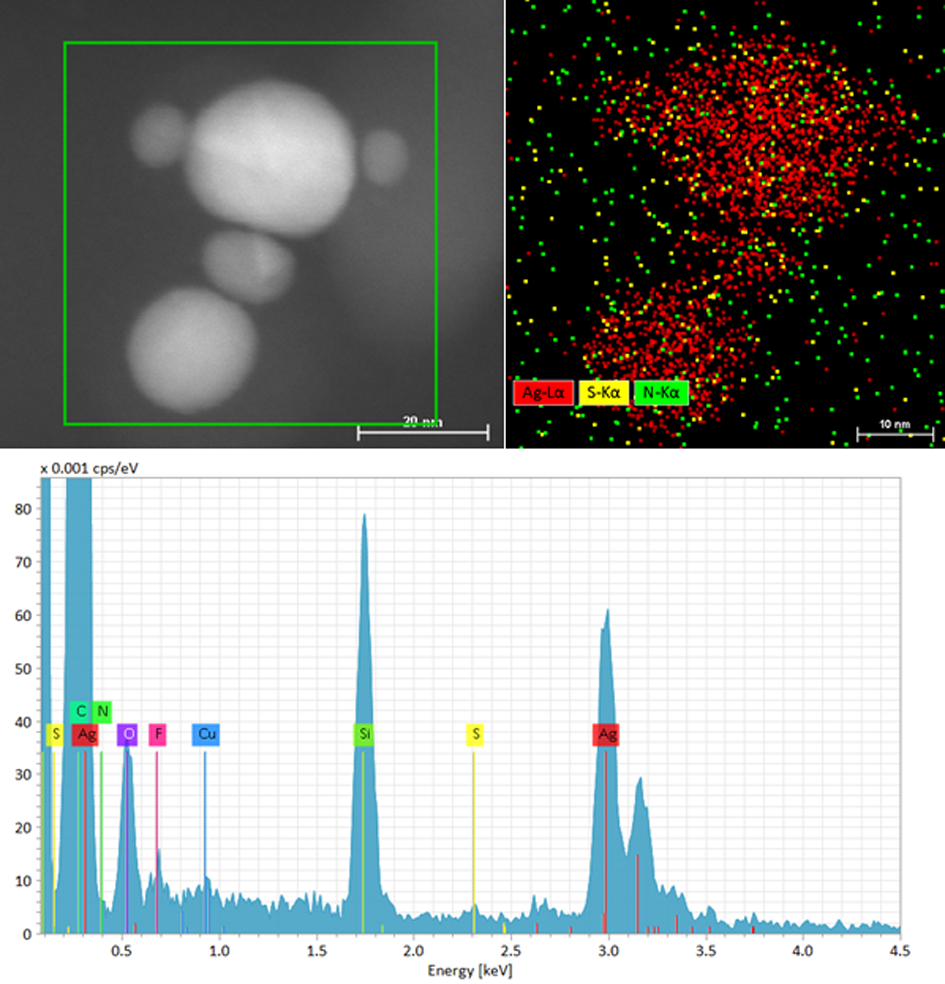


**Supporting Figure 2.** STEM-EDS Map of 3 functionalized AgNps in a small cluster configuration.

**Supporting References**

1. NIST X-ray Photoelectron Spectroscopy Database, Version 4.1 (National Institute of Standards and Technology, Gaithersburg, 2012); http://srdata.nist.gov/xps/
2. Zangmeister, R. A.; Morris, T. A.; Tarlov, M. J. Characterization of Polydopamine Thin Films Deposited at Short Times by Autoxidation of Dopamine, *Langmuir*  **29**, 8619-8628 (2013).
